# Efficient one-step synthesis of diarylacetic acids by electrochemical direct carboxylation of diarylmethanol compounds in DMSO

**DOI:** 10.3762/bjoc.20.203

**Published:** 2024-09-20

**Authors:** Hisanori Senboku, Mizuki Hayama

**Affiliations:** 1 Division of Applied Chemistry, Faculty of Engineering, Hokkaido University, Kita 13 Nishi 8, Kita-ku, Sapporo, Hokkaido 060-8628, Japan; 2 Graduate School of Chemical Sciences and Engineering, Hokkaido University, Kita 13 Nishi 8, Kita-ku, Sapporo, Hokkaido 060-8628, Japan

**Keywords:** C(sp^3^)–O bond cleavage, diarylacetic acid, diarylmethanol, electrochemical reduction, fixation of carbon dioxide

## Abstract

An efficient one-step synthesis of diarylacetic acids was successfully performed by electrochemical direct carboxylation of diarylmethanol compounds in DMSO. Constant-current electrolysis of diarylmethanol species in DMSO using a one-compartment cell equipped with a Pt cathode and a Mg anode in the presence of carbon dioxide induced reductive C(sp^3^)−O bond cleavage at the benzylic position in diarylmethanol compounds and subsequent fixation of carbon dioxide to produce diarylacetic acids in good yield. This protocol provides a novel and simple approach to diarylacetic acids from diarylmethanol species and carbon dioxide without transformation of the hydroxy group into appropriate leaving groups, such as halides and esters including carbonates.

## Introduction

**Scheme 1:**
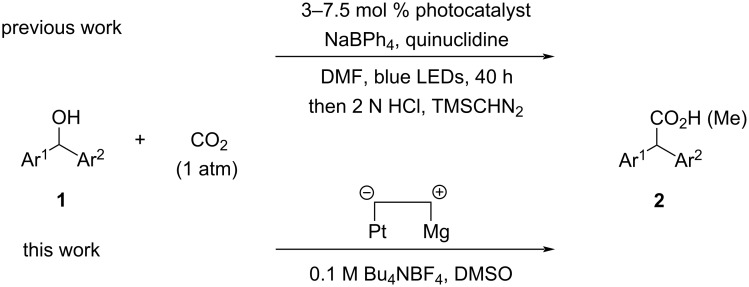
Synthesis of diarylacetic acids **2** from diarylmethanol compounds **1** and carbon dioxide.

Electrochemical reduction of benzyl alcohol derivatives can induce reductive cleavage of a C(sp^3^)–O bond [1] at the benzylic position to generate the corresponding benzylic anion species. This protocol has been frequently applied to electrochemical carboxylation [2–11], yielding phenylacetic acids. For example, Troupel et al. successfully performed electrochemical reduction of benzyl ethers and several esters such as acetate, trifluoroacetate, benzoate, and dibenzyl carbonate derived from benzyl alcohols, including 1-phenylethanol compounds, in the presence of carbon dioxide to give the corresponding phenylacetic acids [12]. We found that alkyl benzyl carbonates and benzal diacetates (benzylidene diacetates) were also applicable to electrochemical carboxylation with C(sp^3^)–O bond cleavage at the benzylic position, yielding phenylacetic acids [13] and mandel acetates [14], respectively. Electrolysis of styrene oxide and related 2-phenylcyclic ethers in the presence of carbon dioxide also induced carboxylation at the benzylic position by reductive cleavage of a C(sp^3^)–O bond to give the corresponding ω-hydroxy-2-phenylalkanoic acids [15–16]. In contrast, little attention has been paid to electrochemical direct carboxylation of benzyl alcohols, although it is a more straightforward and simple protocol toward phenylacetic acids. In 2015, we reported an electrochemical direct carboxylation of benzyl alcohols having an electron-withdrawing group on the phenyl ring [17]. To the best of our knowledge, this is the first report on electrochemical carboxylation of benzyl alcohols. Only benzyl alcohols having an electron-withdrawing group, such as cyano or ester in the *ortho*- or *para*-position of the phenyl ring, were efficiently carboxylated by constant-current electrolysis in DMF using an undivided cell equipped with a Pt cathode and a Mg anode in the presence of carbon dioxide. On the other hand, carboxylation scarcely took place in DMF when other benzyl alcohols were used as substrates. Lundberg and co-workers recently reported similar results showing that electrochemical carboxylation of benzyl alcohols in the presence of Bu_4_NBH_4_ in DMF using graphite electrodes gave the corresponding carboxylic acids only in poor yield [18]. We recently found that DMSO is a more suitable solvent for electrochemical carboxylation of benzyl alcohols. Even though there was no electron-withdrawing group on the phenyl ring in the benzyl alcohols, electrochemical carboxylation of the benzyl alcohols in DMSO provided the corresponding phenylacetic acids in moderate yield by reductive C(sp^3^)–O bond cleavage followed by fixation of carbon dioxide at the benzylic position [19]. Notably, when diphenylmethanol (**1a**) was used as substrate for electrochemical carboxylation in DMSO, the carboxylation efficiently proceeded to give diphenylacetic acid (**2a**) in high yield. In this paper, we focus on electrochemical carboxylation of diarylmethanol compounds **1** in DMSO and report the efficient one-pot synthesis of diarylacetic acids **2** using this protocol (Scheme 1, bottom). Although photochemical synthesis of diarylacetic acids **2** from diarylmethanol species **1** and carbon dioxide has been reported (Scheme 1, top) [20], to the best of our knowledge, this is the first electrochemical and the second efficient example of diarylacetic acid **2** synthesis from diarylmethanol compounds **1** and carbon dioxide in one step.

## Results and Discussion

Although we have previously obtained diphenylacetic acid (**2a**) in 81% yield by electrochemical carboxylation of diphenylmethanol (**1a**) [19], screening of reaction conditions for the substrate **1a** was carried out. Constant-current electrolysis of **1a** using an undivided cell in the presence of carbon dioxide at room temperature was conducted. The effects of the current density (Table 1, entries 1–3), solvent (Table 1, entries 2, 4, and 5), electrodes (Table 1, entries 2, 6, and 7), and electricity (Table 1, entries 2 and 8–10) were investigated. A current density between 10 and 30 mA/cm^2^ had little effect on the yield of **2a** (Table 1, entries 1–3). Carboxylation of **1a** also took place in DMF instead of DMSO as solvent. However, the efficiency and yield were lower than those in DMSO (Table 1, entry 4). Acetonitrile seemed to be unsuitable for the reaction (Table 1, entry 5). Glassy carbon (GC) was usable as cathode material to give **2a** in 59% yield, slightly lower than when using Pt as cathode, probably due to the high hydrogen overpotential (Table 1, entry 6). In contrast, zinc was not effective as an anode material in the carboxylation, probably due to competitive electrochemical reduction of zinc ions generated by electrochemical oxidation of the zinc anode. The deposition of a black precipitate was observed visually at the cathode (Table 1, entry 7). After tuning of the electricity, electrochemical carboxylation of **1a** under the reaction conditions shown in Table 1, entry 8 gave **2a** in 79% isolated yield. In all cases, diphenylmethane, probably produced by protonation of the generated benzyl anion species, was detected as a byproduct in less than 10% yield, determined through ^1^H NMR analysis, except for Table 1, entries 3 (13%) and 5 (25%). It should also be noted that electrolysis was carried out at room temperature, but the temperature of the reaction mixture increased to 40–50 °C at the end of the electrolysis in every case due to heat generation by electric resistance.

**Table 1:** Screening of reaction conditions in the electrochemical carboxylation of **1a**.

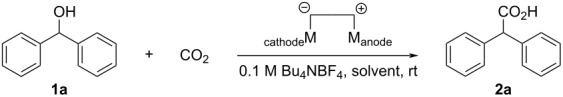							

entry	cathode	anode	solvent	current density, mA/cm^2^	electricity, F/mol	conversion, %^a^	yield, %^b^

1	Pt	Mg	DMSO	10	6	83	69
2	Pt	Mg	DMSO	20	6	84	69
3	Pt	Mg	DMSO	30	6	76	61
4	Pt	Mg	DMF	20	6	48	39
5	Pt	Mg	CH_3_CN	20	6	42	14
6	GC	Mg	DMSO	20	6	71	59
7	Pt	Zn	DMSO	20	6	38	28
8	Pt	Mg	DMSO	20	8	92	79
9	Pt	Mg	DMSO	20	10	94	80
10	Pt	Mg	DMSO	20	12	98	80

^a^Determined by ^1^H NMR spectroscopy using 1,4-dinitrobenzene as internal standard. ^b^Isolated yield.

**Scheme 2:**
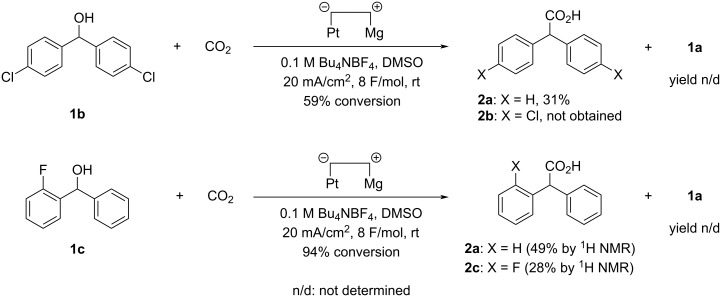
Attempted electrochemical carboxylation of halogen-containing diphenylmethanol compounds **1b** and **1c**.

With these results in hand, the substrate scope was investigated. First, the applicability of halogen-containing diphenylmethanol compounds **1b** and **1c** to the electrochemical carboxylation under the reaction conditions shown in Table 1, entry 8 was investigated, and the results are summarized in Scheme 2. Electrochemical carboxylation of bis(4-chlorophenyl)methanol (**1b**) unfortunately proceeded along with dehalogenation of the phenyl ring to give **2a** in 31% yield. The absence of mono- and dichlorocarboxylic acids such as **2b** in the product and the existence of dechlorinated diphenylmethanol (**1a**) in the recovered starting material were confirmed by ^1^H NMR analysis. These results suggested that **2a** was produced from **1a**, which was generated in situ by electroreductive dechlorination of **1b**. In other words, electroreductive dechlorination of **1b**, producing **1a**, took place preferentially over electroreductive C(sp^3^)–O bond cleavage of **1b**. It should also be noted that no aromatic carboxylic acids were detected by ^1^H NMR analysis. In contrast, the electrochemical carboxylation of **1c**, containing a fluorine atom, gave a mixture of fluorine-containing carboxylic acid **2c** and defluorinated carboxylic acid **2a**. Fluorine-containing starting material **1c** and defluorinated diphenylmethanol (**1a**) were detected by ^1^H NMR analysis. At the same time, it is presently unclear whether **2a** was produced in a carboxylation–defluorination or defluorination–carboxylation sequence.

**Scheme 3:**
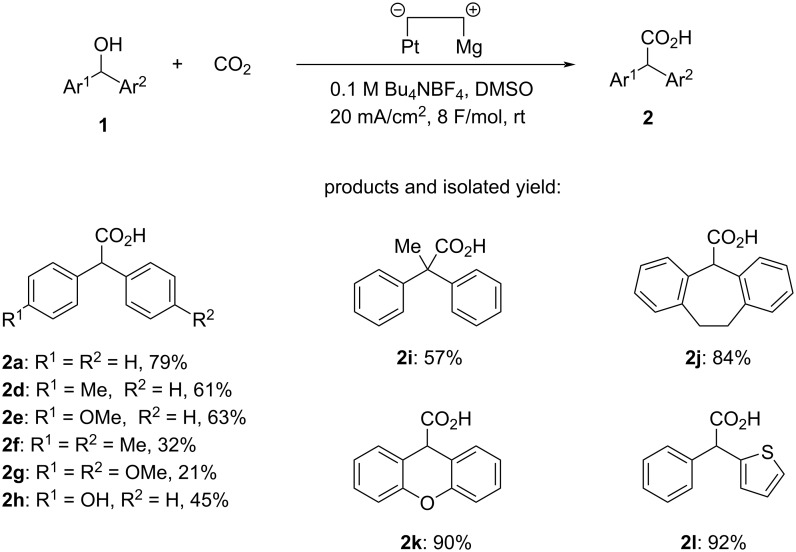
Synthesis of diarylacetic acids **2** by electrochemical carboxylation of diarylmethanol species **1**.

Other results of the electrochemical carboxylation are summarized in Scheme 3. Electrochemical carboxylation of diphenylmethanol species **1d** and **1e**, having a methyl or methoxy group on the phenyl ring, also proceeded to give the corresponding diphenylacetic acids **2d** and **2e** in 61% and 63% yield, respectively. In the electrocarboxylation of **1f** and **1g**, having a methyl or methoxy group on both phenyl rings, the yield of **2f** and **2g** was comparably lower, namely 32% and 21%, respectively, probably due to the electron-donating groups. When diphenylmethanol **1h**, having a hydroxy group on the phenyl ring, was used as substrate, selective C–O bond cleavage, followed by fixation of carbon dioxide, occurred at the benzylic C(sp^3^)–O bond rather than the C(sp^2^)–O bond on the phenyl ring to give the corresponding diphenylacetic acid **2h** in 45% yield. Not only a secondary alcohol but also a tertiary alcohol was applicable to the reaction. When 1,1-diphenylethanol (**1i**) was subjected to electrochemical carboxylation, electroreductive C(sp^3^)–O bond cleavage and subsequent carboxylation also took place, similarly to that of secondary alcohols, to give 2,2-diphenylpropanoic acid (**2i**) in 57% yield. Cyclic alcohols and heteroarylmethanol were also suitable and efficient substrates. Electroreductive C(sp^3^)–O bond cleavage and subsequent carboxylation proceeded efficiently when dibenzocycloheptenol (**1j**) was used as substrate to give the corresponding dibenzocycloheptenecarboxylic acid (**2j**) in 84% yield. Electrocarboxylation of xanthenol (**1k**) provided xanthene-9-carboxylic acid (**2k**) in an excellent yield of 90%. Phenyl(thiophen-2-yl)acetic acid (**2l**) could also be synthesized in an excellent yield of 92% yield by electrochemical carboxylation of phenyl(thiophen-2-yl)methanol (**1l**).

**Scheme 4:**
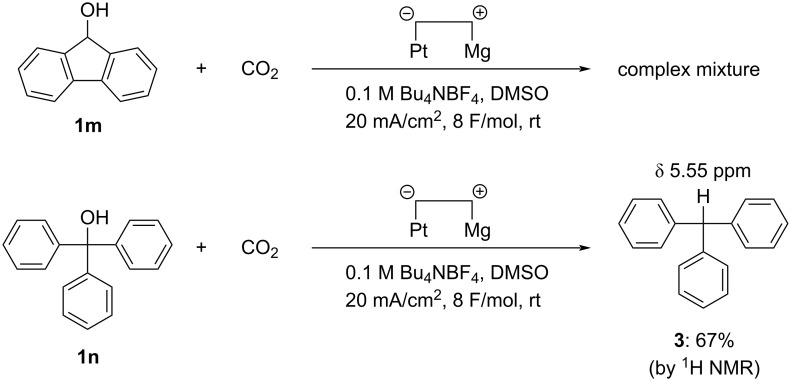
Attempted electrochemical carboxylation of **1m** and **1n**.

Although a wide range of substrates was shown to be applicable, a limitation of the reaction also existed. Electrochemical carboxylation of 9*H*-fluoren-9-ol (**1m**) failed, and only a complex mixture was obtained, probably due to the electrochemical reduction and/or carboxylation of the biphenyl moiety in fluorenol (**1m**, top of Scheme 4). Similarly, when triphenylmethanol (**1n**) was subjected to the electrochemical carboxylation, a small amount of carboxylic acid was obtained as a complex mixture. However, in the ^1^H NMR spectrum of the organic component after extraction with aqueous base, we observed a singlet at δ 5.55 ppm, which could be assigned to the methine proton in triphenylmethane (**3**, Scheme 4, bottom). Further, the yield was determined to be 67% by ^1^H NMR spectroscopy. These results indicated that the triphenylmethyl anion species generated by electroreductive C(sp^3^)–O bond cleavage acted not as nucleophile toward carbon dioxide but as base, performing proton abstraction from DMSO to produce **3** as main product. It was thought that the increase of steric hindrance caused a reduction in nucleophilicity and an increase in basicity in the generated triphenylmethyl anion species, in comparison to the diphenylmethyl anion species.

**Scheme 5:**
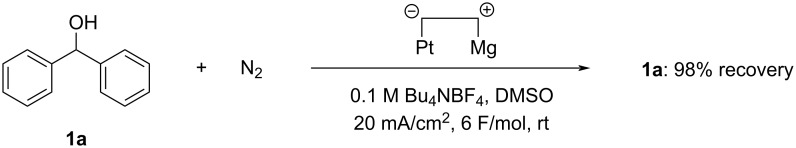
Electrolysis of **1a** under a nitrogen atmosphere.

To elucidate the mechanism of the electroreductive C(sp^3^)–O bond cleavage, one additional experiment was carried out as shown in Scheme 5. Constant-current electrolysis of **1a** in DMSO with 20 mA/cm^2^ of current density and 6 F/mol of electricity at room temperature under a nitrogen atmosphere instead of carbon dioxide resulted in the recovery of 98% of the starting material **1a**. This result indicated that carbon dioxide played an important role not only as a carboxy source but also in the electroreductive C(sp^3^)–O bond cleavage.

**Scheme 6:**
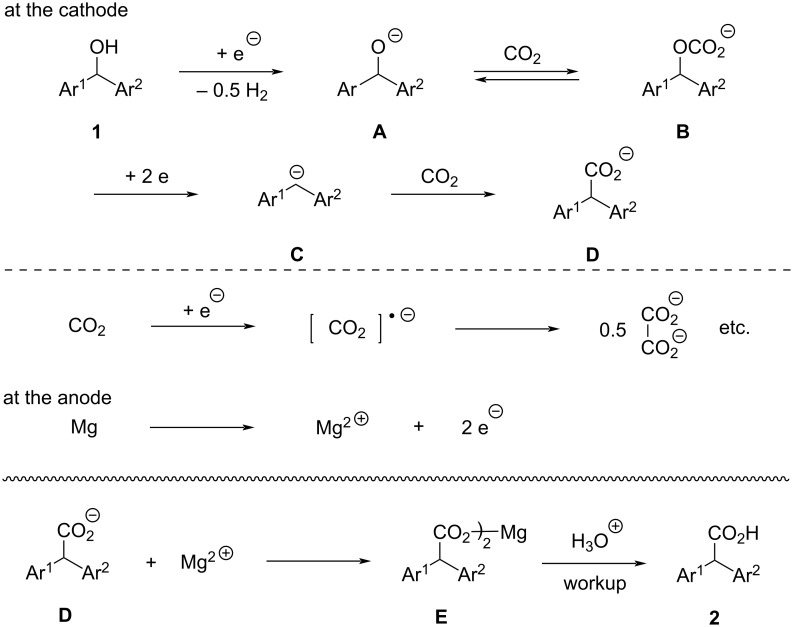
Plausible reaction pathways.

From these and our previous results for the electrochemical carboxylation of benzyl carbonates [13], plausible reaction pathways are proposed as seen in Scheme 6. At the cathode, one-electron reduction of the hydroxy group in diarylmethanol **1** generates H_2_ and the corresponding alkoxide ion **A**, which captures carbon dioxide to form carbonate ion **B**. Although it is currently unclear whether this proceeds in a stepwise or concerted manner, two-electron reduction of carbonate ion **B** generates the corresponding diphenylmethyl anion **C**, which reacts with carbon dioxide to produce a carbon–carbon bond that results in the formation of diphenyl acetate ion **D**. Electrochemical reduction of carbon dioxide competitively occurs at the cathode, and an excess amount of electricity should therefore be necessary to obtain acceptable results. At the anode, on the other hand, dissolution of magnesium ions by electrochemical oxidation of magnesium metal occurs, preventing electrochemical oxidation of the product and intermediates at the anode. The produced acetate ion **D** and a magnesium ion form the salt **E**, which upon acid treatment during workup gives a diphenylacetic acid **2**. The effects of DMSO as solvent are not clear at present, and one reasonable and acceptable aspect might be the solubility of the salt of intermediate **B** in DMSO solvent. Electrochemical reduction of intermediate **B** should occur in solution, and this would mean that intermediate **B** must be dissolved in the solvent used. DMSO is well known as a good solvent for dissolving organic metal salts. In this electrochemical reaction medium, a main counter cation of intermediate **B** is thought to be the magnesium ion, and the magnesium salt of **B** must be dissolved in the solvent. Although other magnesium salts, such as magnesium carbonate and magnesium oxalate, are also generated during the electrolysis, the magnesium salt of **B** would be dissolved sufficiently in DMSO for electrochemical reduction at the cathode.

## Conclusion

Efficient one-pot synthesis of diarylacetic acids **2** was accomplished by electrochemical direct carboxylation of diarylmethanol species **1** in DMSO. 2,2-Diphenylpropanoic acid (**2i**) and phenyl(thiophen-2-yl)acetic acid (**2l**) could also be synthesized from 1,1-diphenylethanol (**1i**) and phenyl(thiophen-2-yl)methanol (**1l**), respectively, in one step by this electrochemical method. Notably, synthesis of xanthene-9-carboxylic acid (**2k**) and dibenzocycloheptene-5-carbocylic acid **2j** was conducted with excellent yield. Direct substitution of a hydroxy by a carboxy group using carbon dioxide as carboxy source is generally difficult under neutral and mild conditions without the use of metal catalysts and/or ligands. This protocol represents a novel method for synthesizing diarylacetic acids **2** from the corresponding alcohols **1** in one step by using an electrochemical method. Instead of DMF and acetonitrile, which have frequently been used for electrochemical carboxylation, DMSO was found to be effective for electrochemical carboxylation of diarylmethanol compounds **1** in order to provide diarylacetic acids **2**. The present electrochemical synthesis is promising as a novel, efficient, facile, and green organic method.

## Experimental

### General information

^1^H (400 MHz) and ^13^C (100 MHz) NMR spectra were recorded in CDCl_3_ or DMSO-*d*_6_ with a JEOL JNM-ECS400 FT NMR spectrometer. The chemical shifts δ are given in ppm with tetramethylsilane (δ 0 ppm) or DMSO (δ 2.50 ppm) for ^1^H and CDCl_3_ (δ 77.0 ppm) or DMSO-*d*_6_ (δ 39.5 ppm) for ^13^C as internal references. *J* values are in Hz. Peak multiplicities are given as follows: s, singlet; d, doublet; t, triplet; q, quartet; m, multiplet; br, broad. Reagents and solvents, including anhydrous DMSO, are commercially available and were used as received without further purification. Electrochemical reactions were carried out using a constant current power supply (model 5944), Metronix Corp., Tokyo.

Diphenylmethanol (**1a**) [21], bis(4-chlorophenyl)methanol (**1b**) [22], (2-fluorophenyl)phenylmethanol (**1c**) [23], (4-methylphenyl)phenylmethanol (**1d**) [21], (4-methoxyphenyl)phenylmethanol (**1e**) [21], bis(4-methylphenyl)methanol (**1f**) [24], bis(4-methoxyphenyl)methanol (**1g**) [25], (4-hydroxyphenyl)phenylmethanol (**1h**) [26], 10,11-dihydro-5*H*-dibenzo[*a*,*d*]cyclohepten-5-ol (**1j**) [27], 9*H*-xanthen-9-ol (**1k**) [28], and phenyl(thiophen-2-yl)methanol (**1l**) [21] are known compounds and were prepared from the corresponding commercially available ketones by reaction with NaBH_4_ in EtOH or EtOH/THF 1:1. 1,1-Diphenylethanol (**1i**) is also a known compound and was prepared according to the reported procedure [29]. 9*H*-Fluoren-9-ol (**1m**) and triphenylmethanol (**1n**) are commercially available.

### General procedure for electrochemical carboxylation of **1**

A test-tube-like (≈25 mm ⌀) undivided cell equipped with a Pt plate cathode (2 × 2 cm^2^), a Mg rod anode (6 mm ⌀, 2 cm), and a Teflon^®^ tube (1 mm ⌀) for supplying carbon dioxide were used for the electrolysis. A solution of a diarylmethanol **1** (1 mmol) in anhydrous DMSO (10 mL) containing 0.1 M Bu_4_NBF_4_ (329 mg, 1 mmol) was set in the cell under a nitrogen atmosphere. Carbon dioxide was bubbled through the solution at room temperature for 10 min, and then the solution was electrolyzed with a constant current (20 mA/cm^2^) under atmospheric pressure of bubbling carbon dioxide at room temperature. The temperature of the reaction mixture increased to 40–50 °C at the end of the electrolysis in every case due to generation of heat by electric resistance during the electrolysis. After 8 F/mol of electricity had been supplied, 1 M hydrochloric acid (100 mL) was added to the electrolyzed solution, and then the mixture was extracted with ethyl acetate (30 mL × 5). The combined organic layer was washed with aqueous saturated NaHCO_3_ (40 mL × 3), and the resulting aqueous solution was acidified with 3 M hydrochloric acid and then extracted with ethyl acetate (30 mL × 5). The combined ethyl acetate solution was washed with H_2_O (100 mL × 3) and dried over MgSO_4_. Evaporation of the solvent gave a diarylacetic acid **2**. The organic layer, extracted by aqueous saturated NaHCO_3_, was washed with H_2_O (100 mL × 3) and dried over MgSO_4_. Evaporation of the solvent gave a residue that was analyzed by ^1^H NMR spectroscopy, during which 1,4-dinitrobenzene was used as an internal standard for quantification of the substances.

The products diphenylacetic acid (**2a**) [30–31], (4-methylphenyl)phenylacetic acid (**2d**) [32], (4-methoxyphenyl)phenylacetic acid (**2e**) [32], bis(4-methylphenyl)acetic acid (**2f**) [30,32], bis(4-methoxyphenyl)acetic acid (**2g**) [30,33], (4-hydroxyphenyl)phenylacetic acid (**2h**) [34], 2,2-diphenylpropaoic acid (**2i**) [35], 10,11-dihydro-5*H*-dibenzo[*a*,*d*]cycloheptene-5-carboxylic acid (**2j**) [30], 9*H*-xanthene-9-carboxylic acid (**2k**) [36], and phenyl(thiophen-2-yl)acetic acid (**2l**) [30] are known compounds, and their spectral data were good agreement with previously reported values.

### Spectral data of the products **2**

Diphenylacetic acid (**2a**): ^1^H NMR (400 MHz, CDCl_3_, δ) 5.05 (s, 1H), 7.25–7.34 (m, 10H); ^13^C NMR (100 MHz, CDCl_3_, δ) 56.9, 127.5, 128.7 (× 2), 137.8, 178.1.

(4-Methylphenyl)phenylacetic acid (**2d**): ^1^H NMR (400 MHz, CDCl_3_, δ) 2.32 (s, 3H), 5.01 (s, 1H), 7.14 (d, *J* = 8.2 Hz, 2H), 7.21 (d, *J* = 8.2 Hz, 2H), 7.25–7.29 (m, 1H), 7.31–7.32 (m, 4H); ^13^C NMR (100 MHz, CDCl_3_, δ) 21.0, 56.6, 127.4, 128.5, 128.59, 128.61, 129.3, 134.9, 137.2, 138.0, 178.9.

(4-Methoxyphenyl)phenylacetic acid (**2e**): ^1^H NMR (400 MHz, CDCl_3_, δ) 3.78 (s, 3H), 4.99 (s, 1H), 6.86 (d, *J* = 8.7 Hz, 2H), 7.24 (d, *J* = 8.7 Hz, 2H), 7.23–7.32 (m, 5H); ^13^C NMR (100 MHz, CDCl_3_, δ) 55.2, 56.1, 114.0, 127.4, 128.5, 128.6, 129.7, 129.9, 138.3, 159.0, 179.2.

Bis(4-methylphenyl)acetic acid (**2f**): ^1^H NMR (400 MHz, CDCl_3_, δ) 2.32 (s, 6H), 4.97 (s, 1H), 7.13 (d, *J* = 8.2 Hz, 4H), 7.20 (d, *J* = 8.2 Hz, 4H); ^13^C NMR (100 MHz, CDCl_3_, δ) 21.0, 56.2, 128.5, 129.3, 135.1, 137.1, 178.9.

Bis(4-methoxyphenyl)acetic acid (**2g**): ^1^H NMR (400 MHz, CDCl_3_, δ) 3.78 (s, 6H), 4.95 (s, 1H), 6.86 (d, *J* = 8.7 Hz, 4H), 7.23 (d, *J* = 8.7 Hz, 4H); ^13^C NMR (100 MHz, CDCl_3_, δ) 55.2 (OMe and CHCO_2_H), 114.0, 129.6, 130.3, 158.9, 179.2.

(4-Hydroxyphenyl)phenylacetic acid (**2h**): ^1^H NMR (400 MHz, DMSO-*d*_6_, δ) 4.91 (s, 1H), 6.70 (d, *J* = 8.7 Hz, 2H), 7.10 (d, *J* = 8.7 Hz, 2H), 7.20–7.36 (m, 5H), 9.35 (s, 1H); ^13^C NMR (100 MHz, DMSO-*d*_6_, δ) 56.0, 115.8, 127.2, 128.8, 128.9, 130.0, 130.2, 140.1, 156.8, 174.3.

2,2-Diphenylpropanoic acid (**2i**): ^1^H NMR (400 MHz, CDCl_3_, δ) 1.94 (s, 3H), 7.26–7.34 (m, 10H); ^13^C NMR (100 MHz, CDCl_3_, δ) 26.8, 56.4, 127.0, 128.06, 128.12, 143.7, 181.3.

10,11-Dihydro-5*H*-dibenzo[*a*,*d*]cycloheptene-5-carboxylic acid (**2j**): ^1^H NMR (400 MHz, CDCl_3_, δ) 2.85–2.93 (m, 2H), 3.31–3.39 (m, 2H), 4.81 (s, 1H), 7.14–7.24 (m, 8H); ^13^C NMR (100 MHz, CDCl_3_, δ) 32.4, 58.7, 126.3, 128.1, 130.4, 131.3, 135.4, 139.6, 178.2.

9*H*-Xanthene-9-carboxylic acid (**2k**): ^1^H NMR (400 MHz, CDCl_3_, δ) 4.97 (s, 1H), 7.06–7.10 (m, 2H), 7.12–7.15 (m, 2H), 7.28–7.32 (m, 4H); ^13^C NMR (100 MHz, CDCl_3_, δ) 44.9, 117.0, 117.6, 123.4, 129.1, 129.3, 151.3, 177.1.

Phenyl(thiophen-2-yl)acetic acid (**2l**): ^1^H NMR (400 MHz, CDCl_3_, δ) 5.24 (s, 1H), 6.95–6.97 (m, 1H), 7.01–7.02 (m, 1H), 7.24–7.25 (m, 1H), 7.29–7.37 (m, 3H), 7.39–7.42 (m, 2H); ^13^C NMR (100 MHz, CDCl_3_, δ) 52.1, 125.5, 126.6, 126.7, 128.0, 128.3, 128.8, 137.6, 140.0, 177.8.

## Supporting Information

File 1 NMR spectra.

## Data Availability

All data that supports the findings of this study is available in the published article and/or the supporting information to this article.
