# Supplementary material for: Efficient one-step synthesis of diarylacetic acids by electrochemical direct carboxylation of diarylmethanol compounds in DMSO
Source: Beilstein J Org Chem. 2024 Sep 20;20:2392–400. doi: 10.3762/bjoc.20.203 (PMC11420549; doi:10.3762/bjoc.20.203)
Supplement: File 1 — NMR spectra. [file Beilstein_J_Org_Chem-20-2392-s001.pdf]

BEILSTEIN JOURNAL OF ORGANIC CHEMISTRY

Supporting Information

for

Efficient one-step synthesis of diarylacetic acids by electrochemical direct carboxylation of diarylmethanol compounds in DMSO

Hisanori Senboku and Mizuki Hayama

Beilstein J. Org. Chem. **2024,** *20,* 2392–2400. doi:10.3762/bjoc.20.203

NMR spectra

License and Terms: This is a supporting information file under the terms of the Creative Commons Attribution License (https://creativecommons.org/licenses/by/4.0). Please note that the reuse, redistribution and reproduction in particular requires that the author(s) and source are credited and that individual graphics may be subject to special legal provisions.
The license is subject to the *Beilstein Journal of Organic Chemistry* terms and conditions: (https://www.beilstein-journals.org/bjoc/terms)

Table of Contents:

1. ^{1}H and ^{13}C Spectra of compound **2a** S2
2. ^{1}H and ^{13}C Spectra of compound **2d** S4
3. ^{1}H and ^{13}C Spectra of compound **2e** S6
4. ^{1}H and ^{13}C Spectra of compound **2f** S8
5. ^{1}H and ^{13}C Spectra of compound **2g** S10
6. ^{1}H and ^{13}C Spectra of compound **2h** S12
7. ^{1}H and ^{13}C Spectra of compound **2i** S14
8. ^{1}H and ^{13}C Spectra of compound **2j** S16
9. ^{1}H and ^{13}C Spectra of compound **2k** S18
10. ^{1}H and ^{13}C Spectra of compound **2l** S20

1. ^{1}H and ^{13}C spectra of compound 2a.

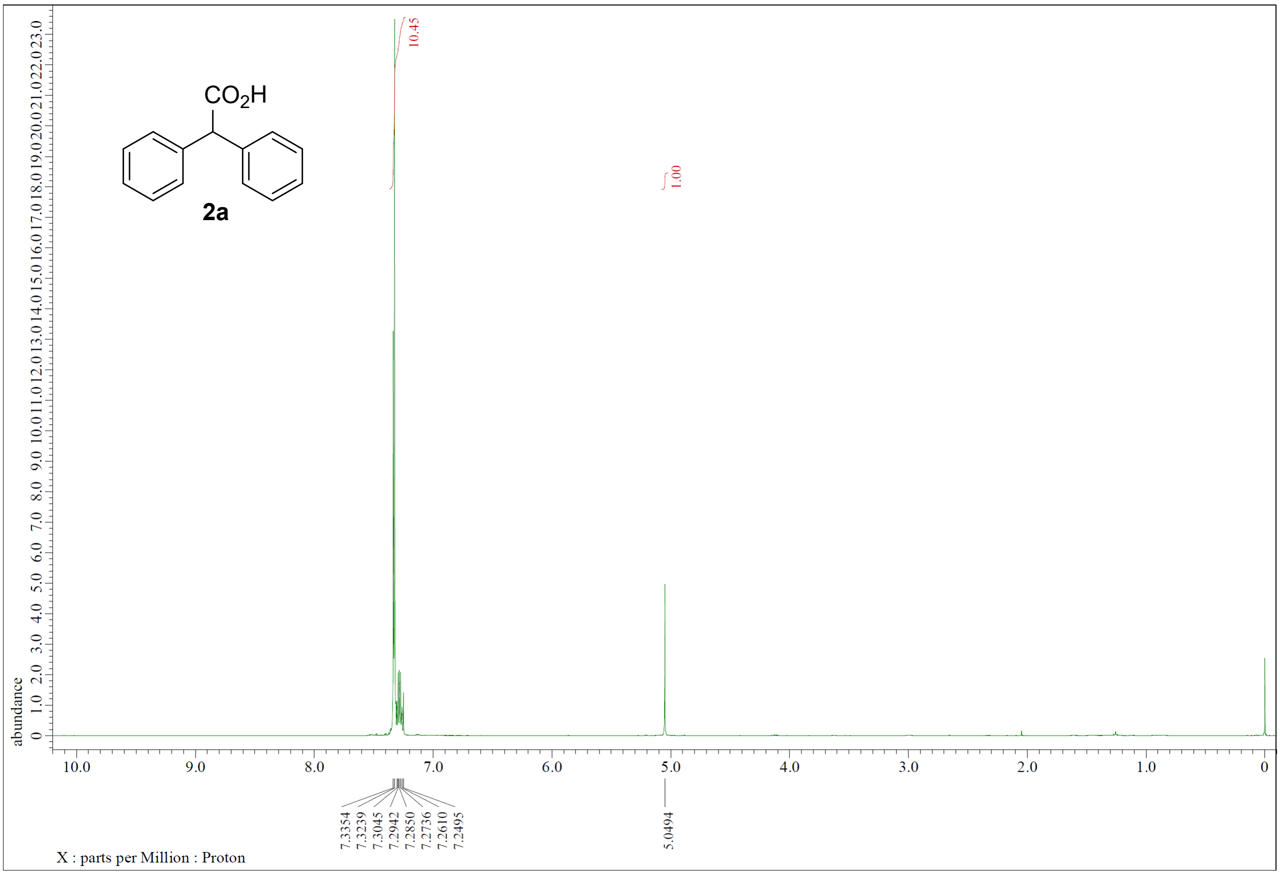

Fig. 1. ^{1}H NMR (400 MHz; $CDCl_3$) spectrum of diphenylacetic acid (**2a**).

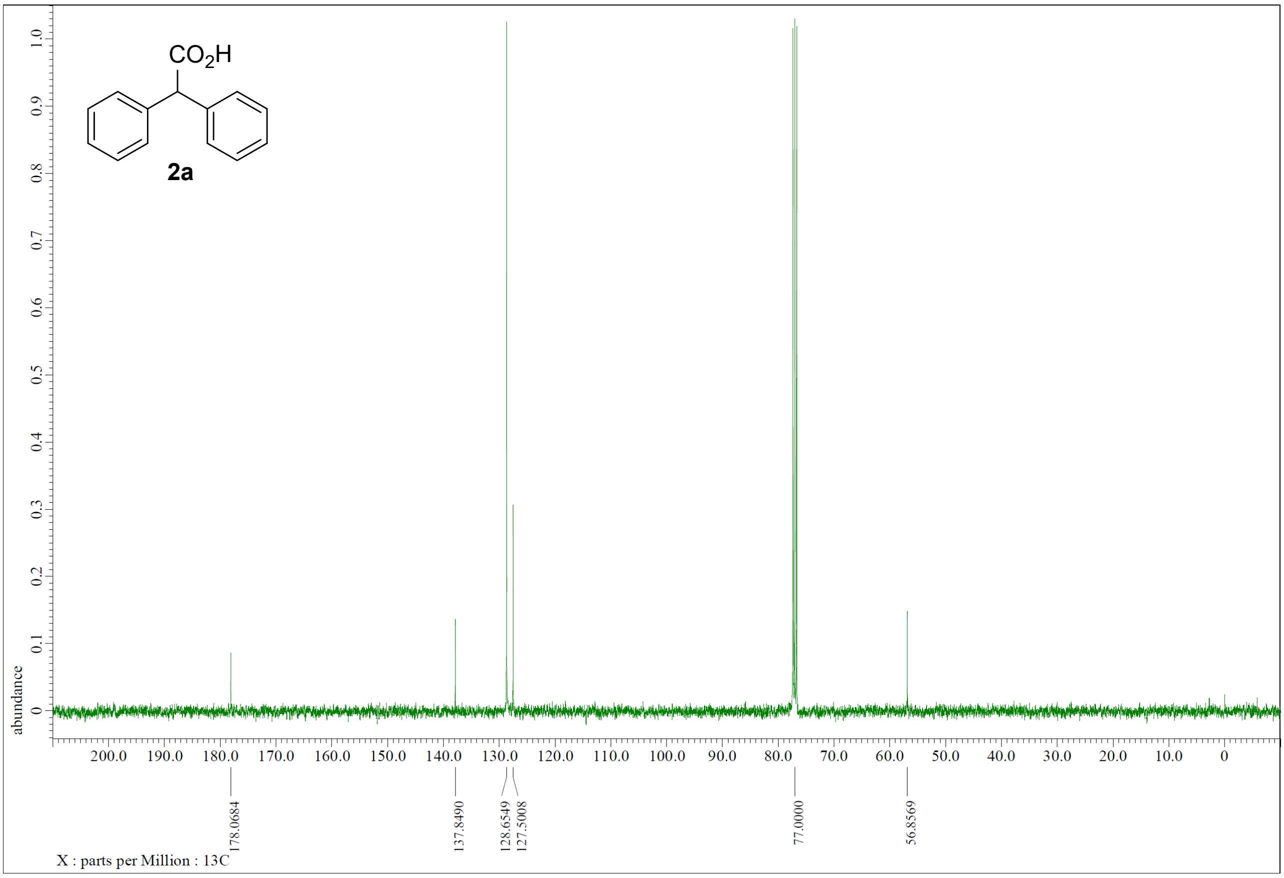

Fig. 2. ^{13}C NMR (100 MHz; $CDCl_3$) spectrum of diphenylacetic acid (**2a**).

2. ^{1}H and ^{13}C spectra of compound 2d.

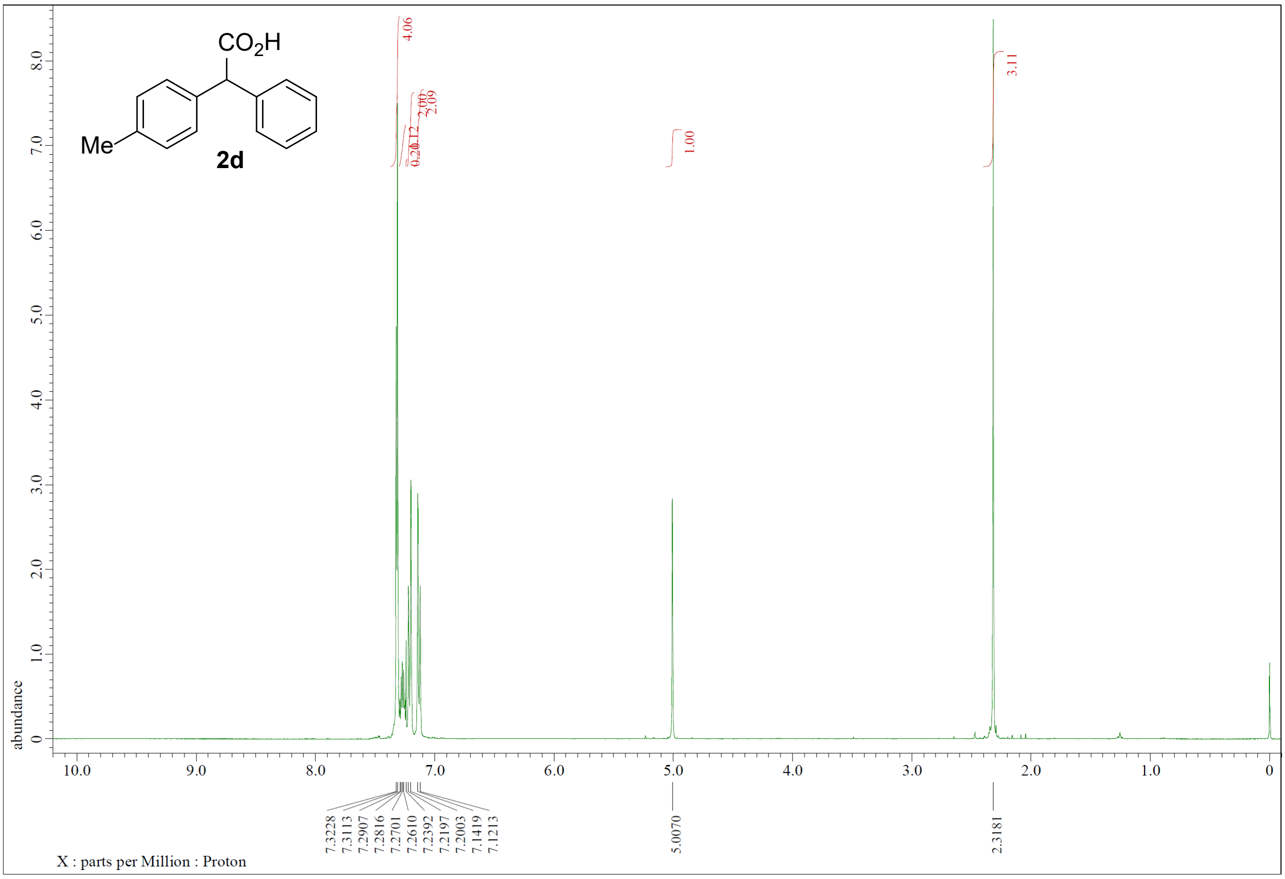

Fig. 3. ^{1}H NMR (400 MHz; $CDCl_3$) spectrum of (4-methylphenyl)phenylacetic acid (**2d**).

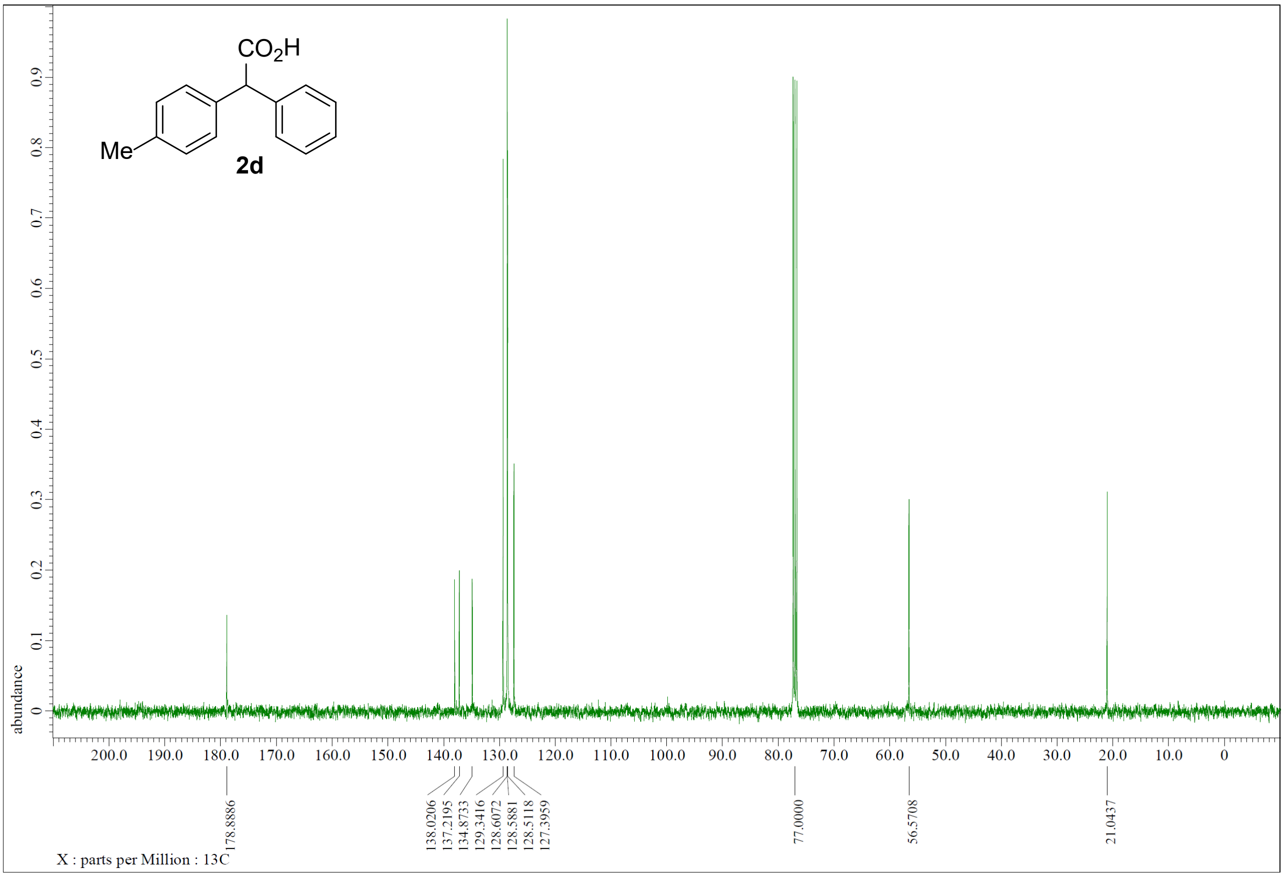

Fig. 4. ^{13}C NMR (100 MHz; $CDCl_3$) spectrum of (4-methylphenyl)phenylacetic acid (**2d**).

3. ^{1}H and ^{13}C spectra of compound 2e.

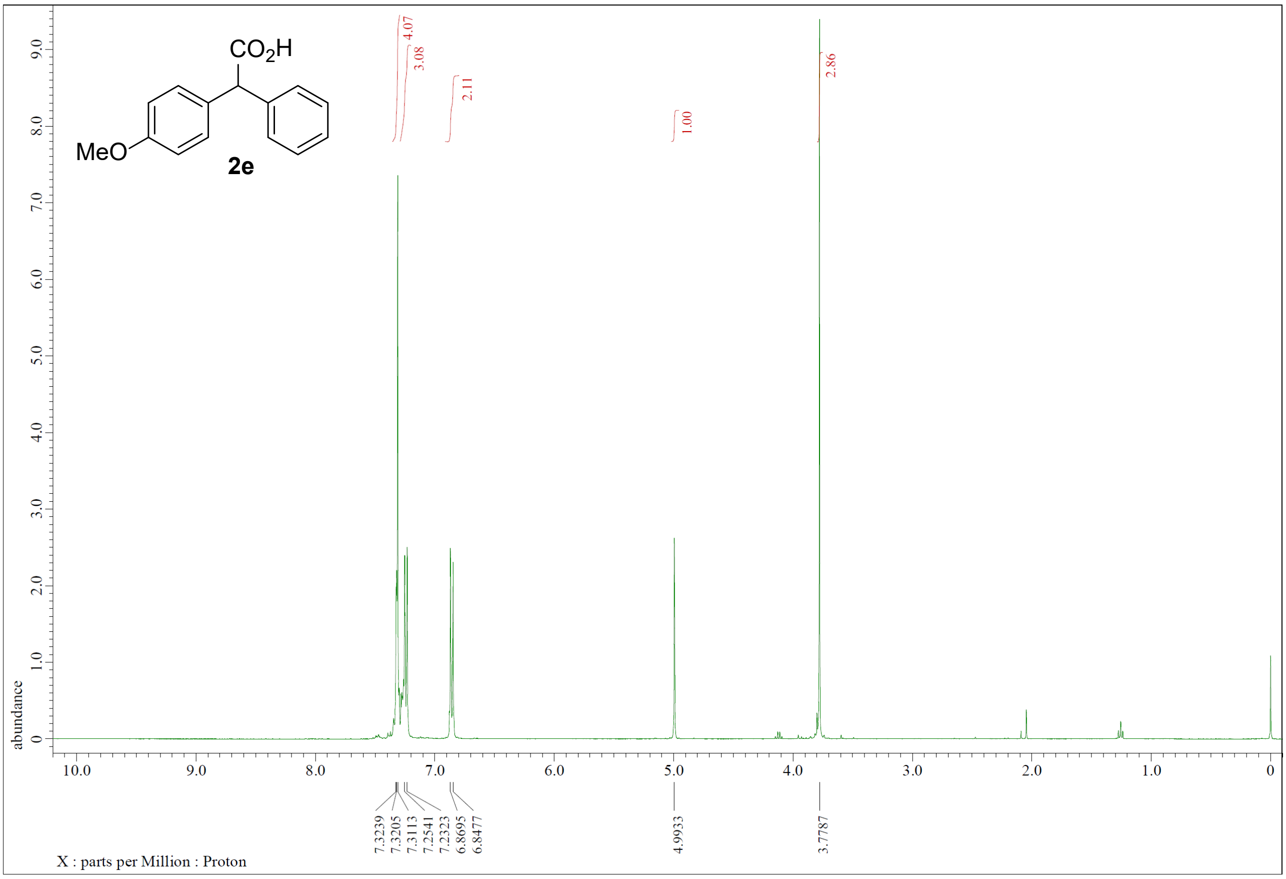

Fig. 5. ^{1}H NMR (400 MHz; $CDCl_3$) spectrum of (4-methoxylphenyl)phenylacetic acid (**2e**).

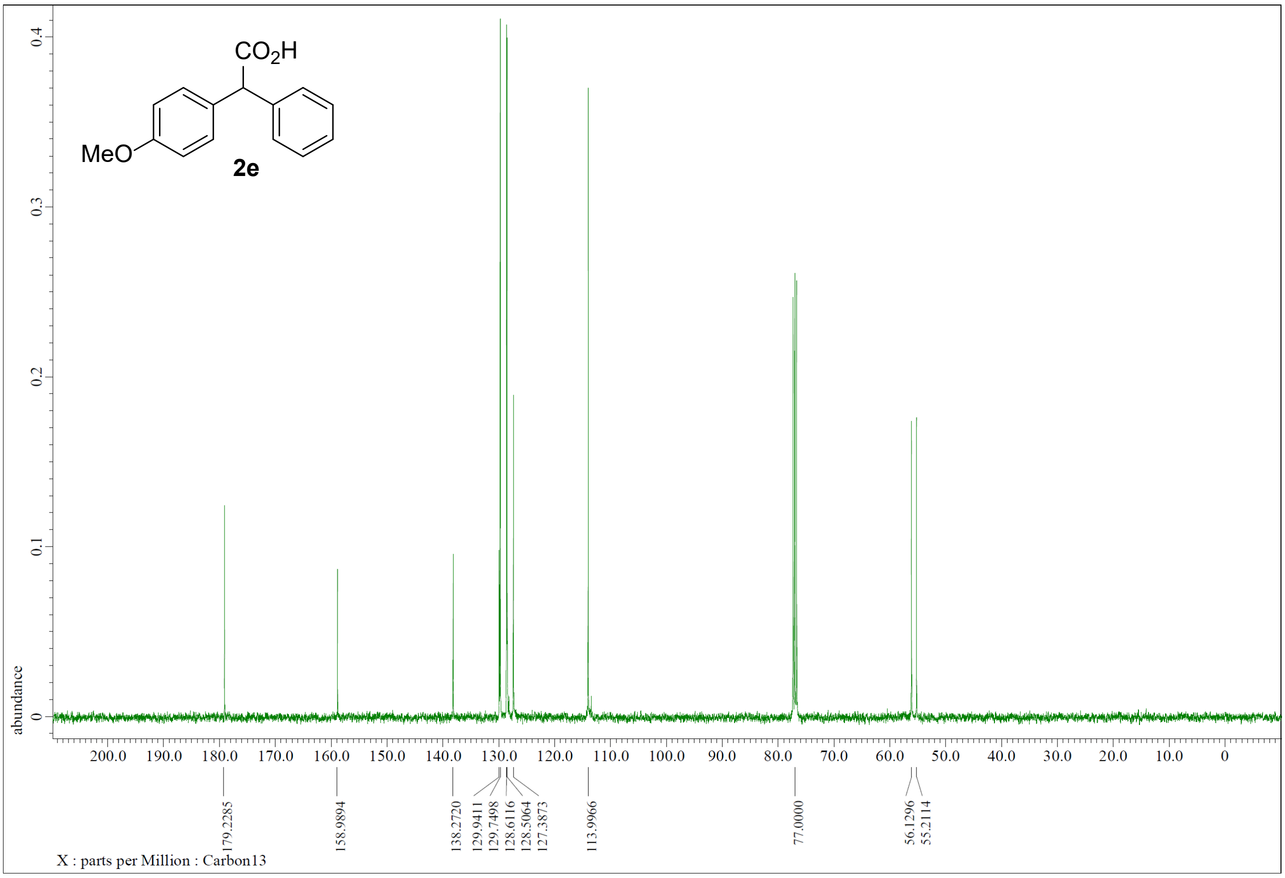

Fig. 6. ^{13}C NMR (100 MHz; $CDCl_3$) spectrum of (4-methoxylphenyl)phenylacetic acid (**2e**).

4. ^{1}H and ^{13}C spectra of compound 2f.

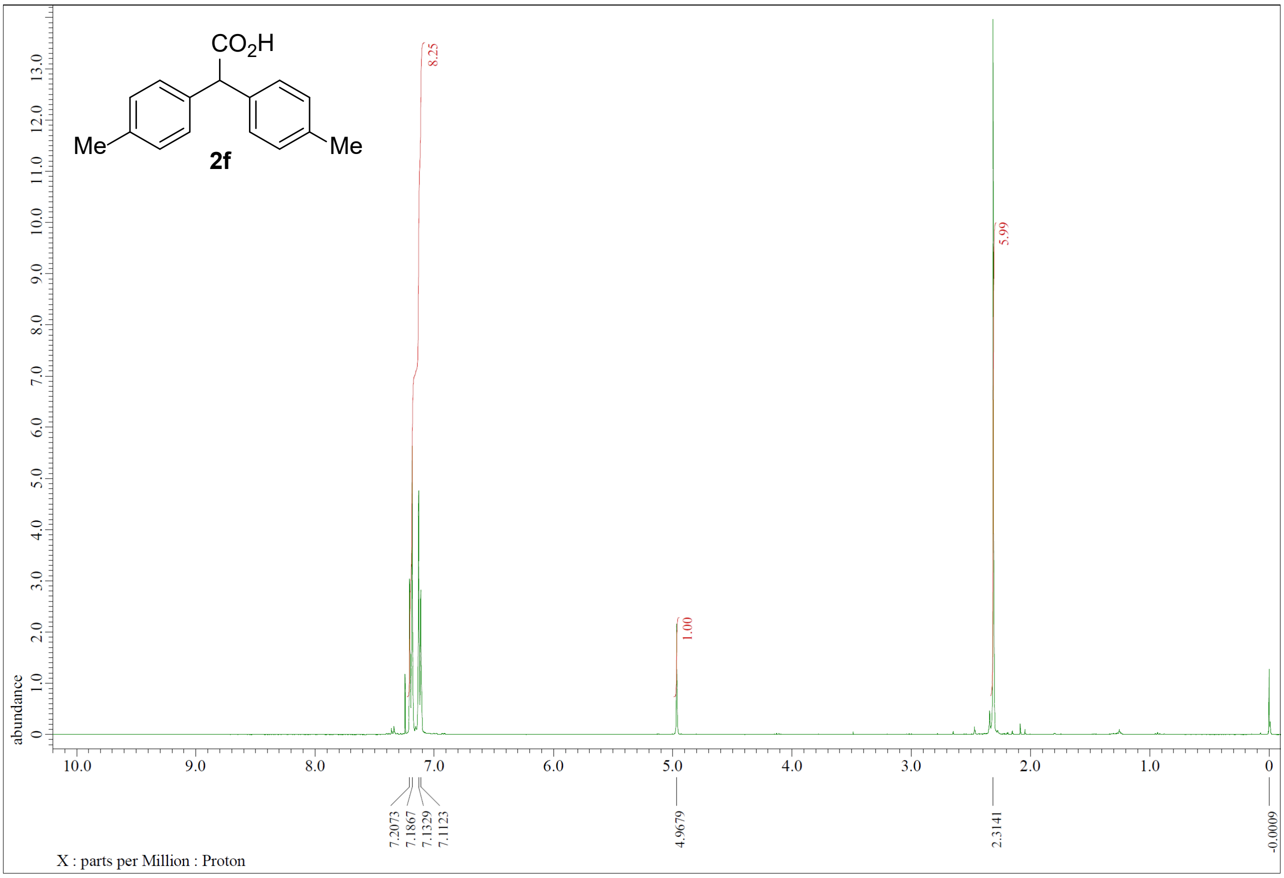

Fig. 7. ^{1}H NMR (400 MHz; $CDCl_3$) spectrum of bis(4-methylphenyl)acetic acid (**2f**).

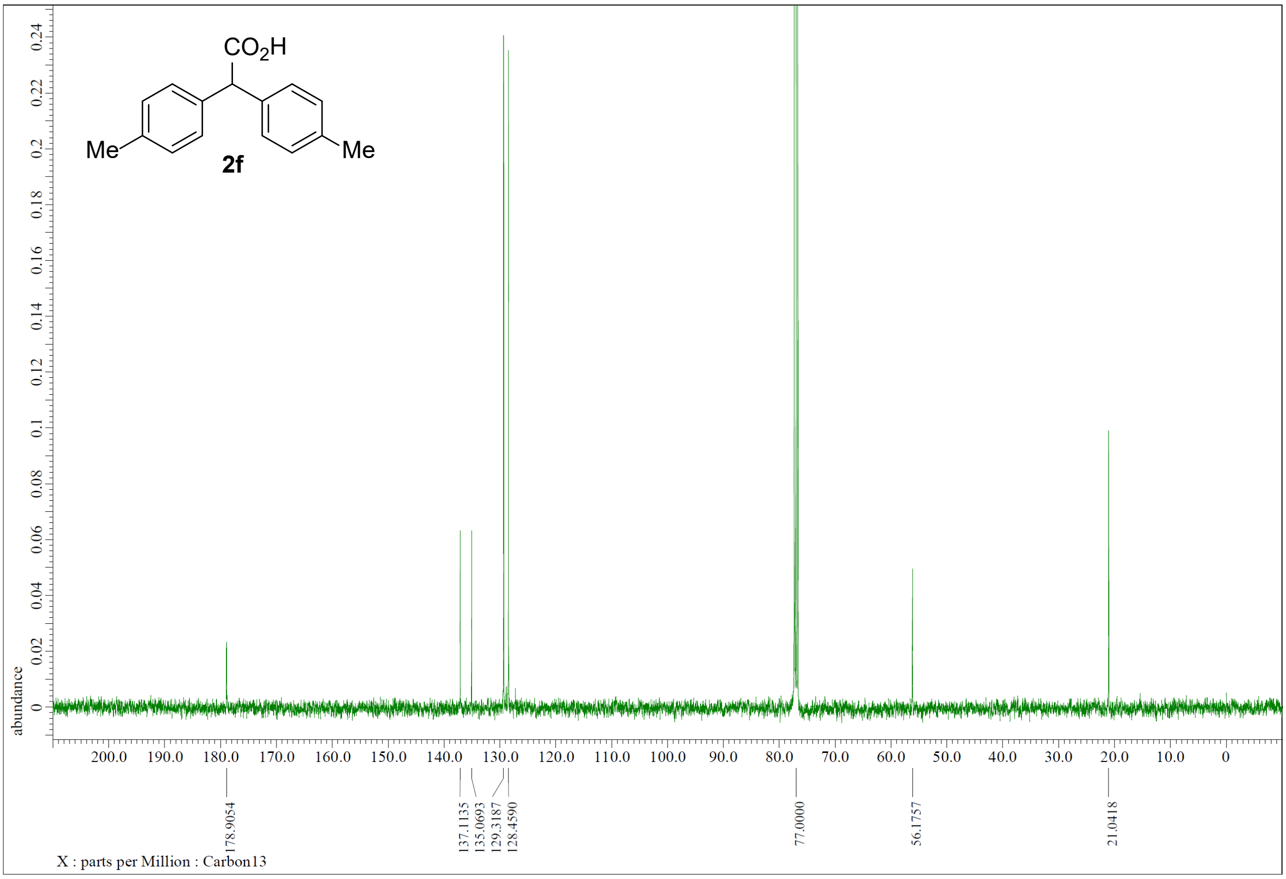

Fig. 8. ^{13}C NMR (100 MHz; $CDCl_3$) spectrum of bis(4-methylphenyl)acetic acid (**2f**).

5. ^1H and ^{13}C spectra of compound 2g.

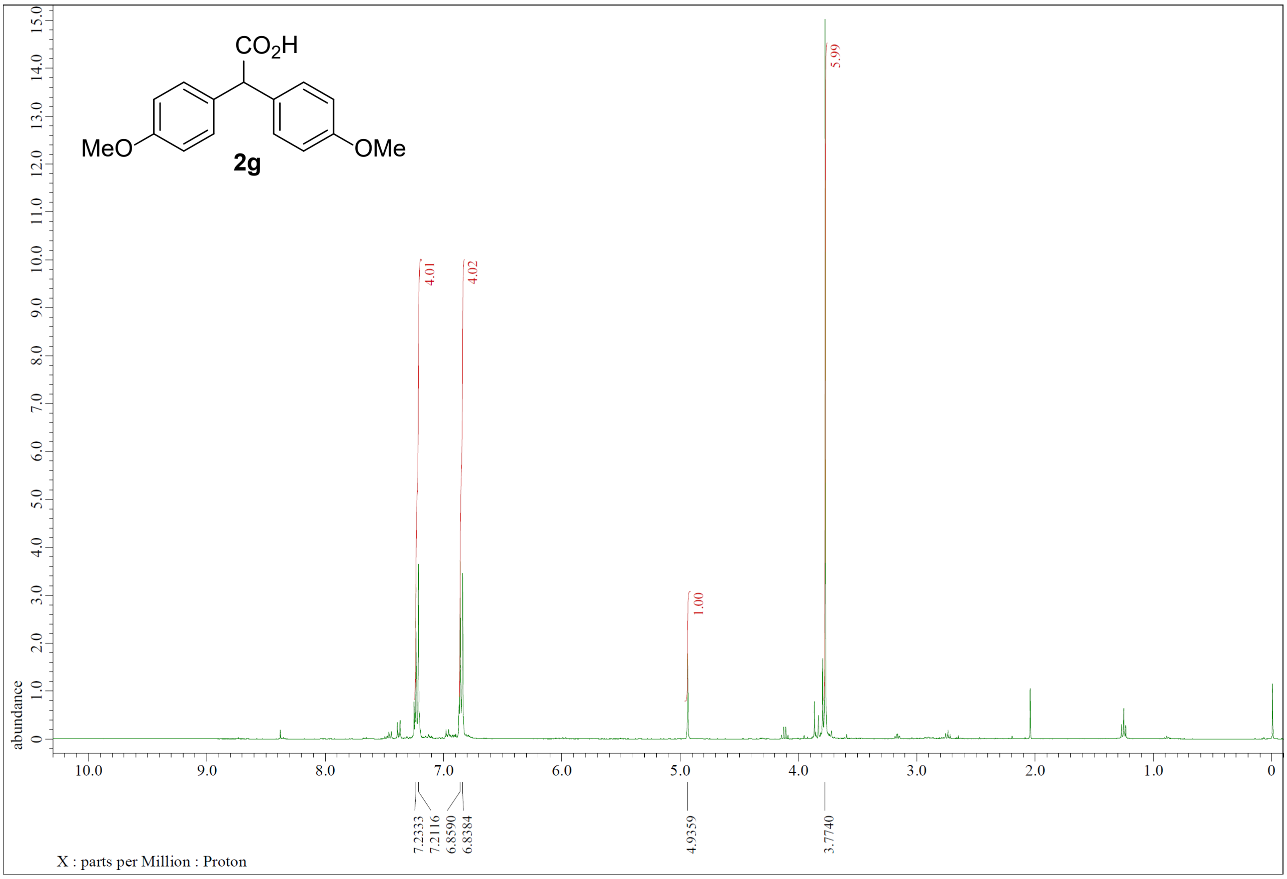

Fig. 9. ^1H NMR (400 MHz; $CDCl_3$) spectrum of bis(4-methoxylphenyl)acetic acid (**2g**).

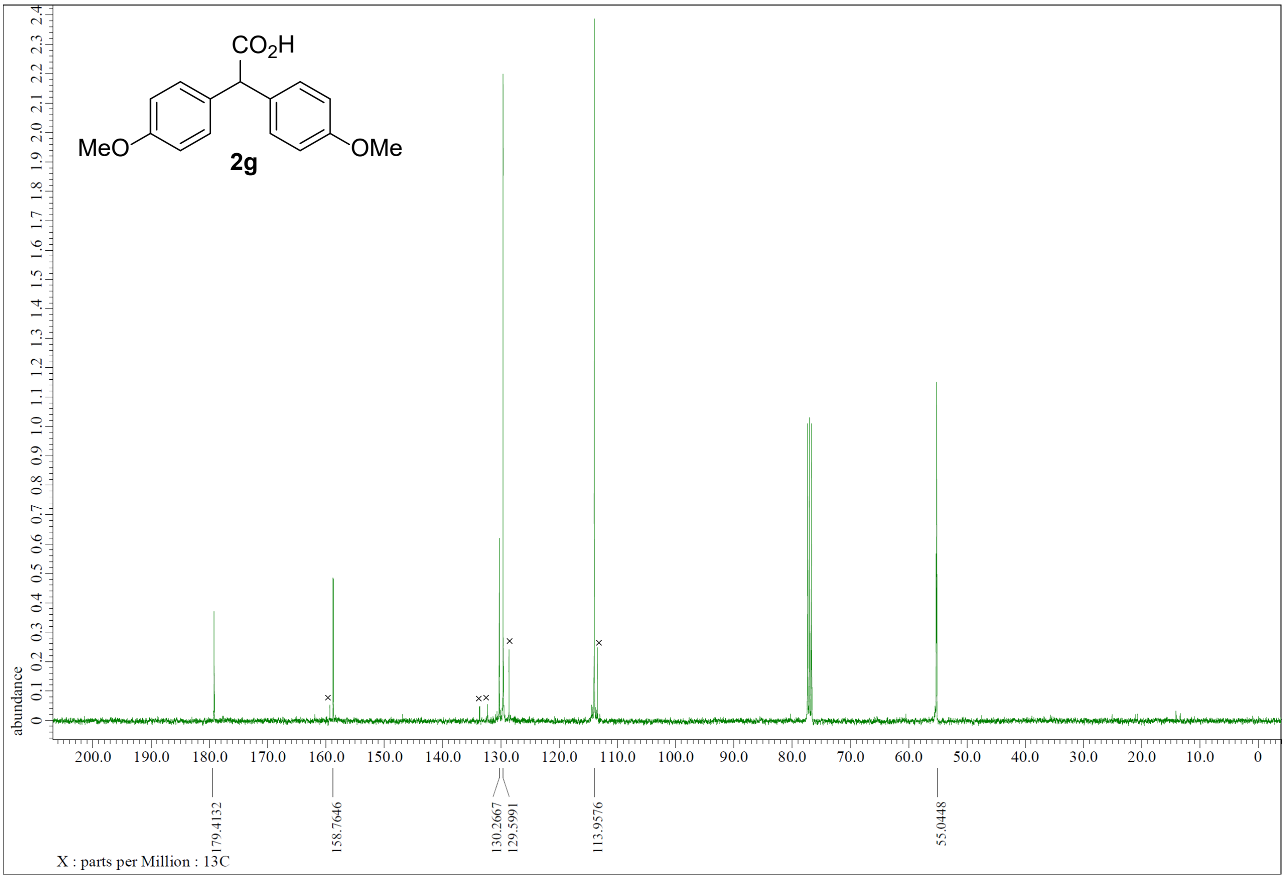

Fig. 10. ^{13}C NMR (100 MHz; $CDCl_3$) spectrum of bis(4-methoxylphenyl)acetic acid (**2g**).

6. ^{1}H and ^{13}C spectra of compound 2h.

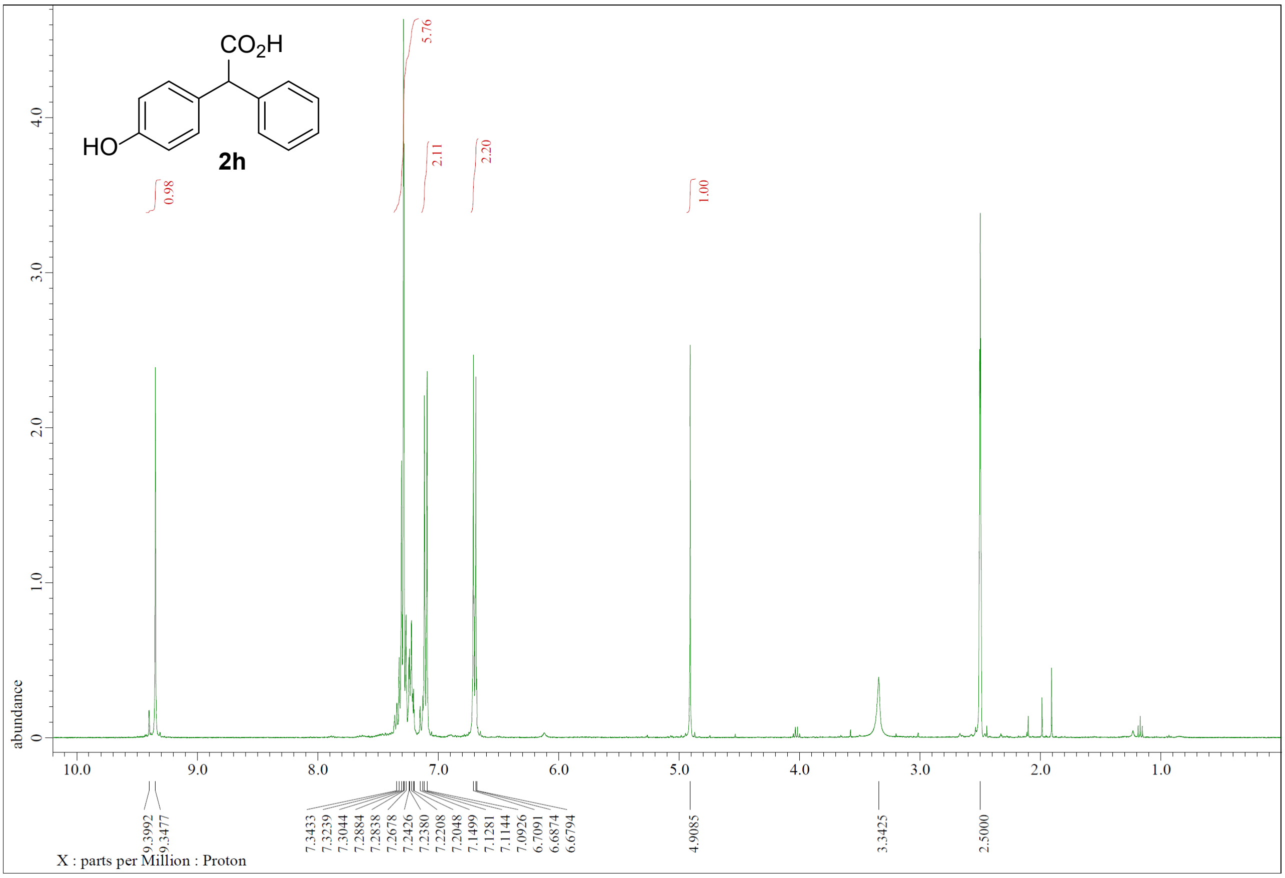

Fig. 11. ^{1}H NMR (400 MHz; DMSO-d6) spectrum of (4-hydroxyphenyl)phenylacetic acid (**2h**).

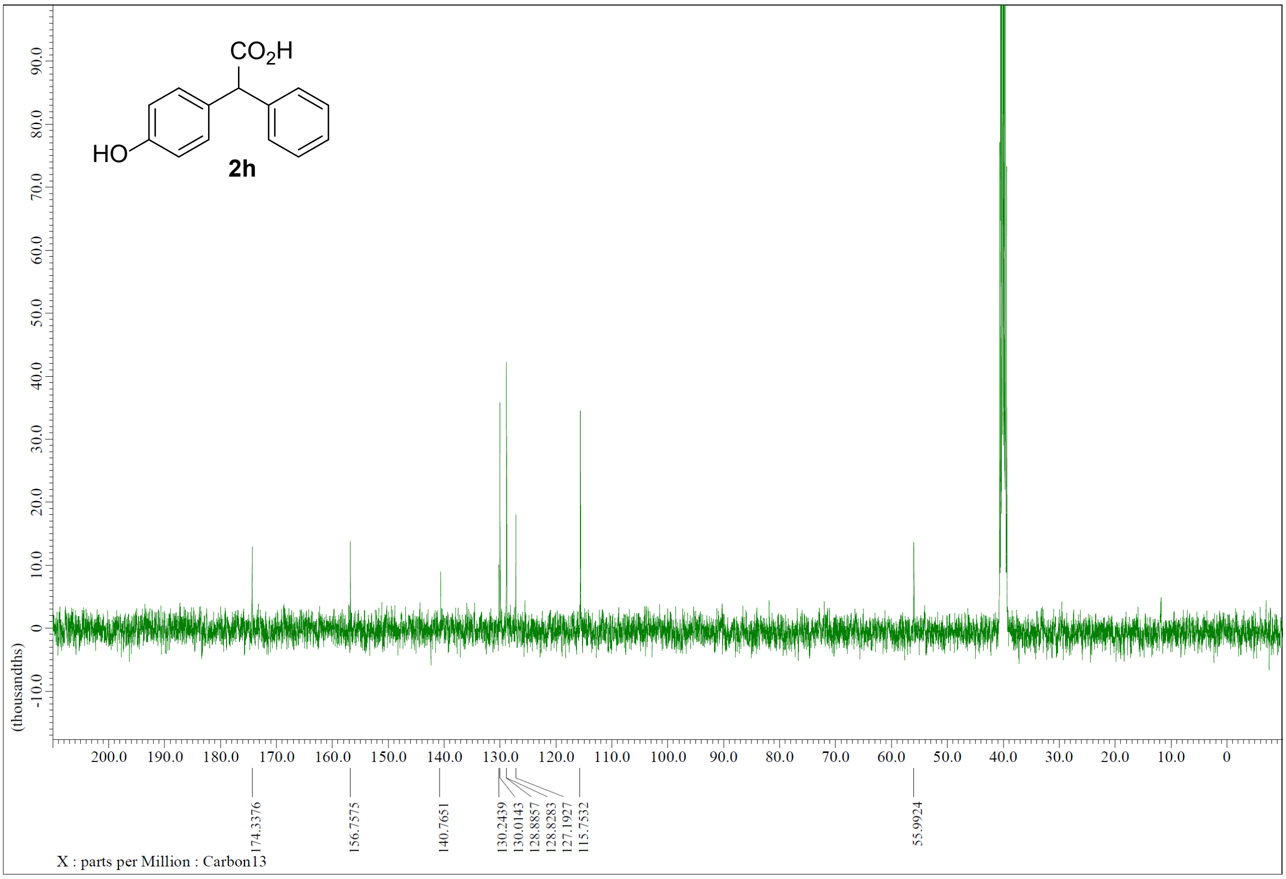

Fig. 12. ^{13}C NMR (100 MHz; DMSO-d6) spectrum of (4-hydroxyphenyl)phenylacetic acid (**2h**).

7. ^{1}H and ^{13}C spectra of compound 2i.

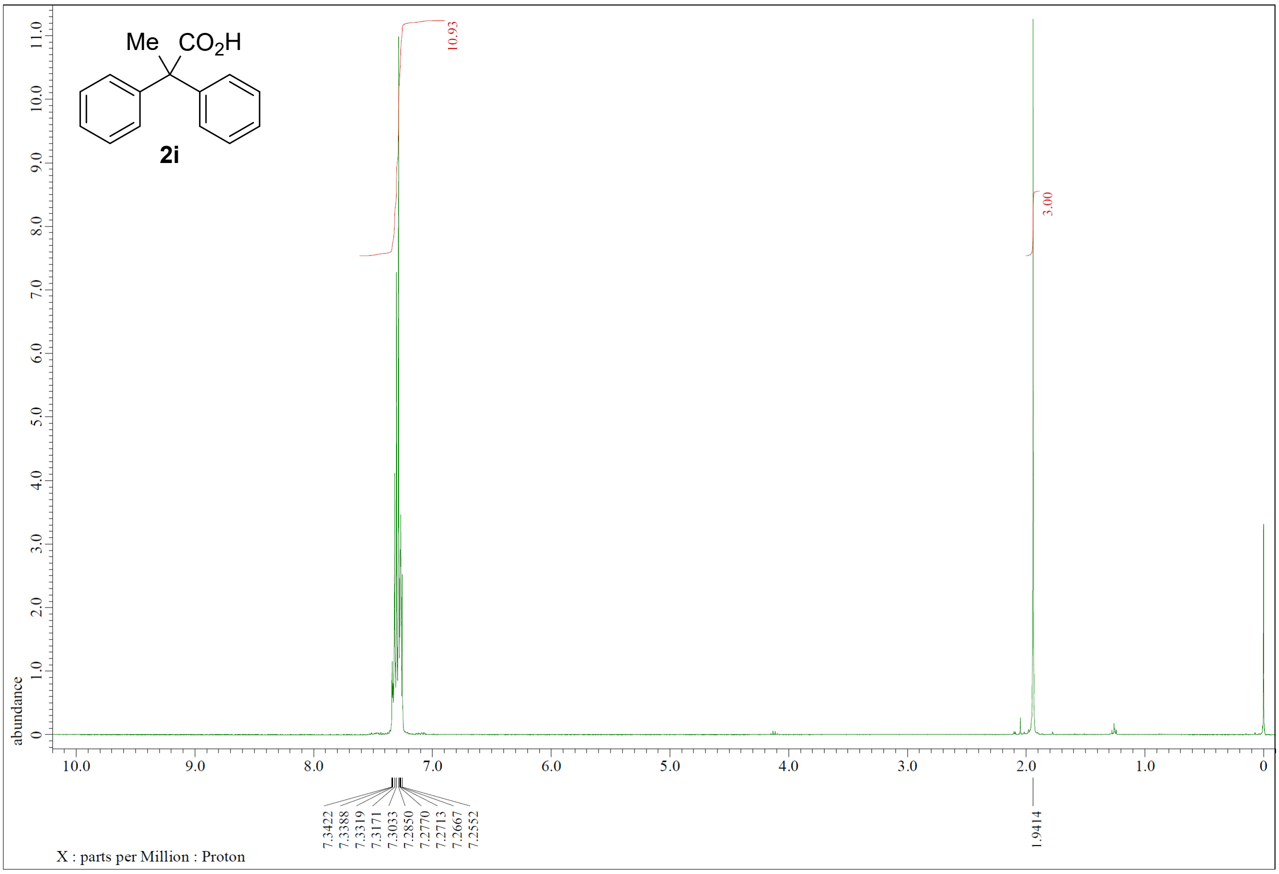

Fig. 13. ^{1}H NMR (400 MHz; $CDCl_3$) spectrum of 2,2-diphenylpropanoic acid (**2i**).

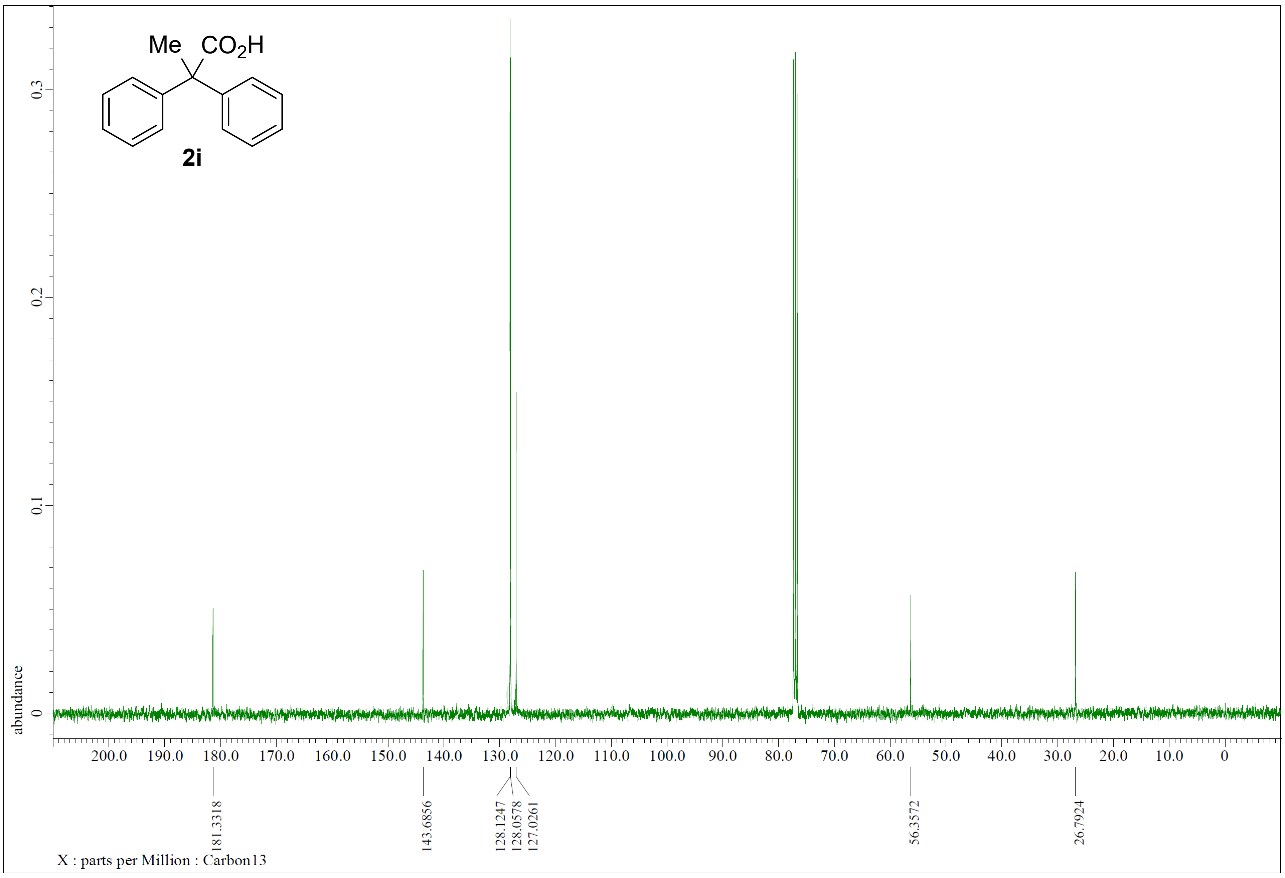

Fig. 14. ^{13}C NMR (100 MHz; $CDCl_3$) spectrum of 2,2-diphenylpropanoic acid (**2i**).

8. ^{1}H and ^{13}C spectra of compound 2j.

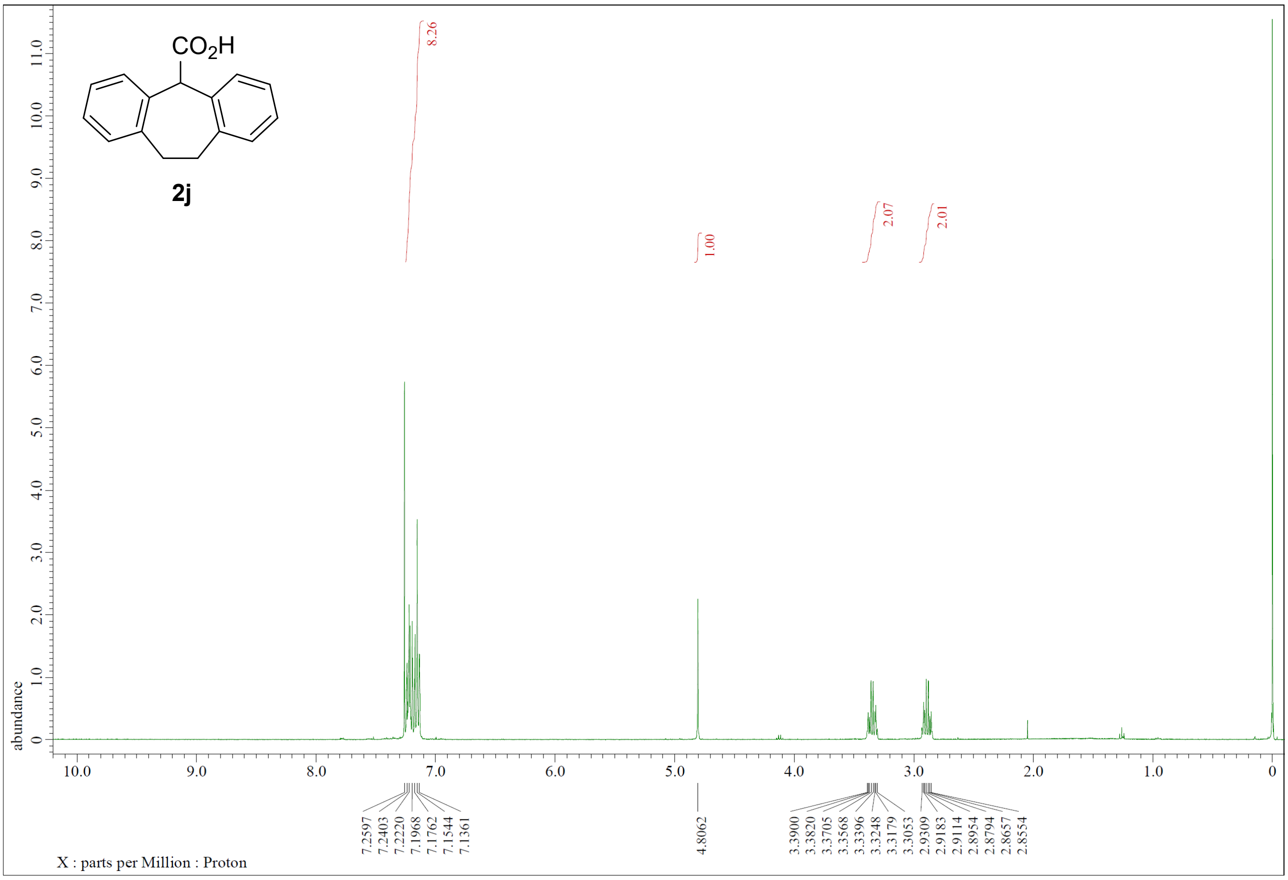

Fig. 15. ^{1}H NMR (400 MHz; $CDCl_3$) spectrum of 10,11-dihydro-5H-dibenzo[a,d]cycloheptene-5-carboxylic acid (**2j**).

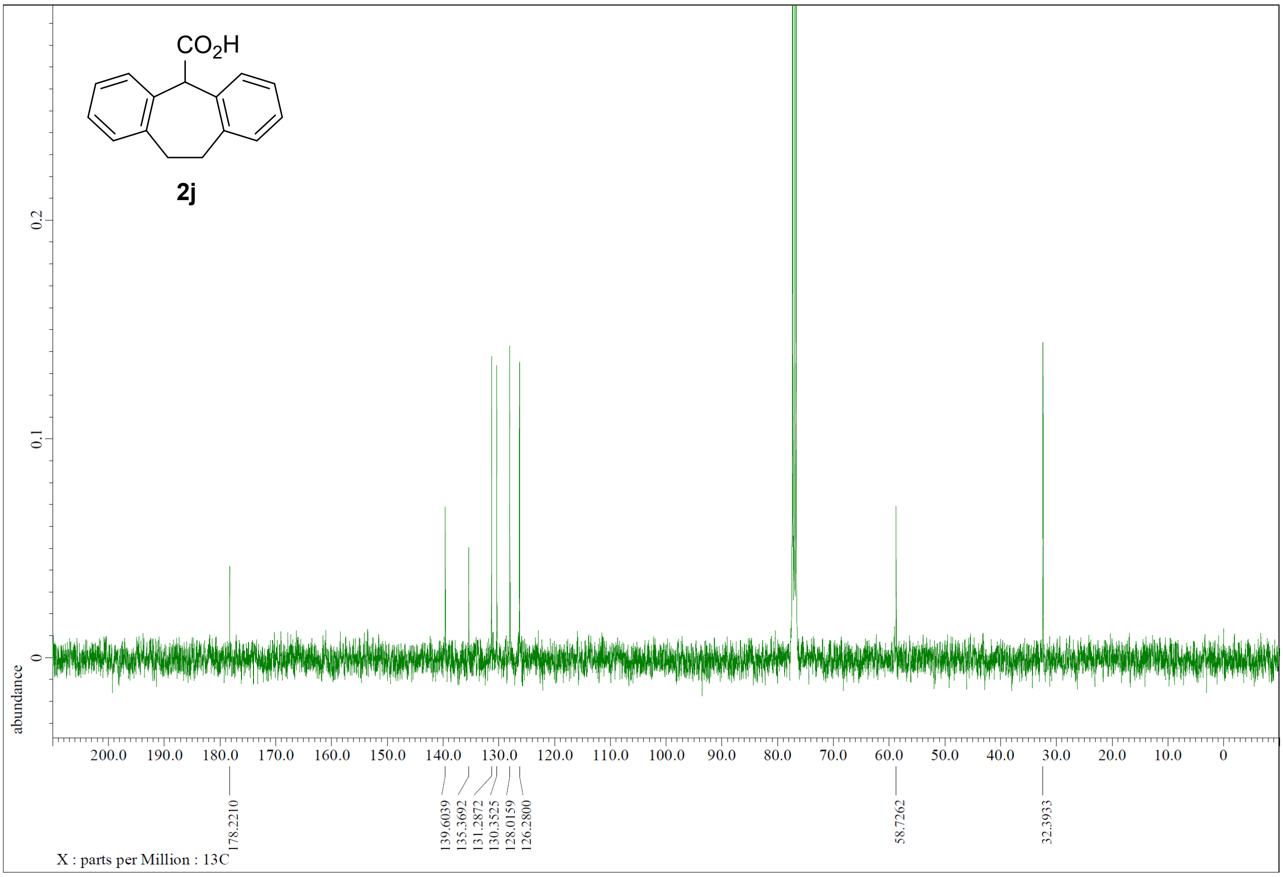

Fig. 16. ^{13}C NMR (100 MHz; $CDCl_3$) spectrum of 10,11-dihydro-5H-dibenzo[a,d]cycloheptene-5-carboxylic acid (**2j**).

9. ^1H and ^{13}C spectra of compound 2k.

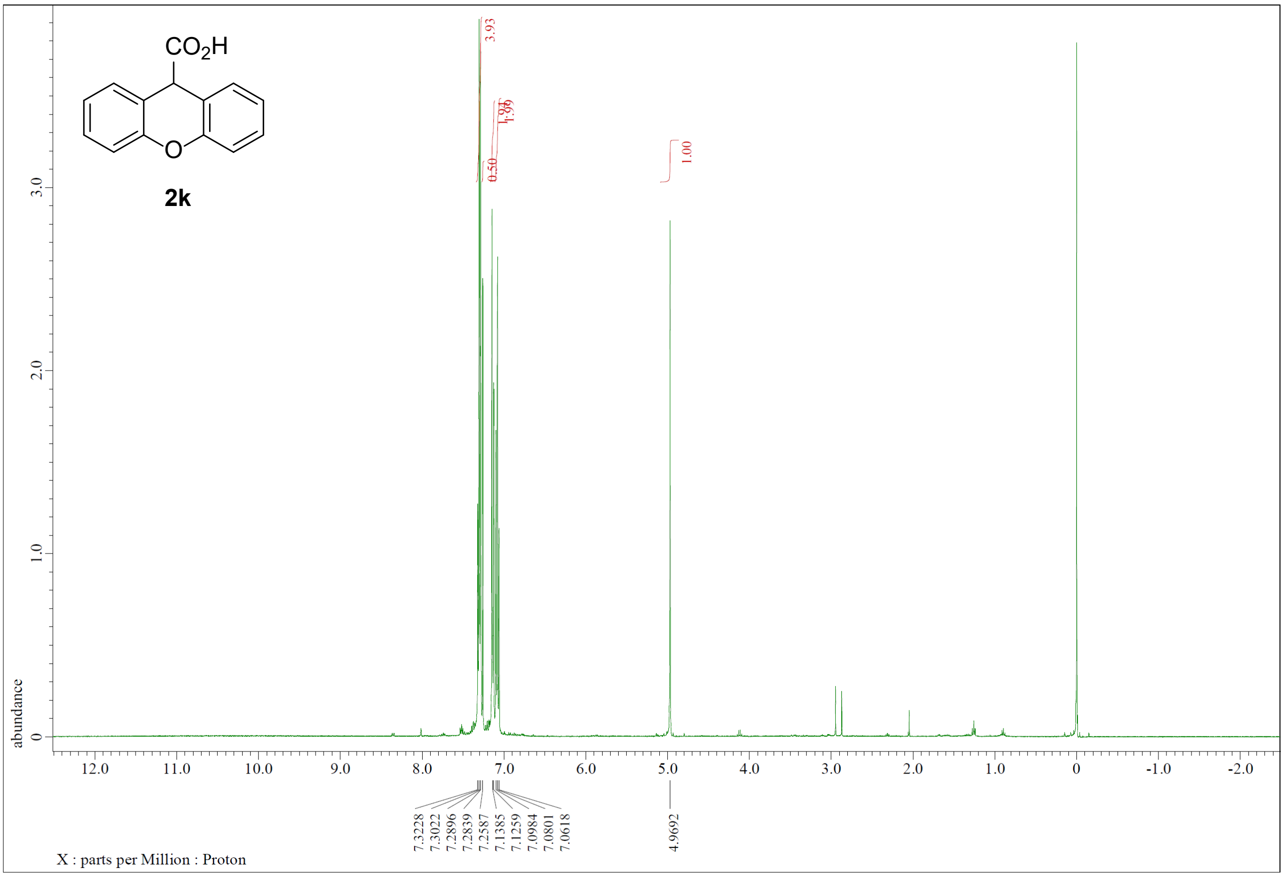

Fig. 17. ^1H NMR (400 MHz; $CDCl_3$) spectrum of 9H-xanthene-9-carboxylic acid (**2k**).

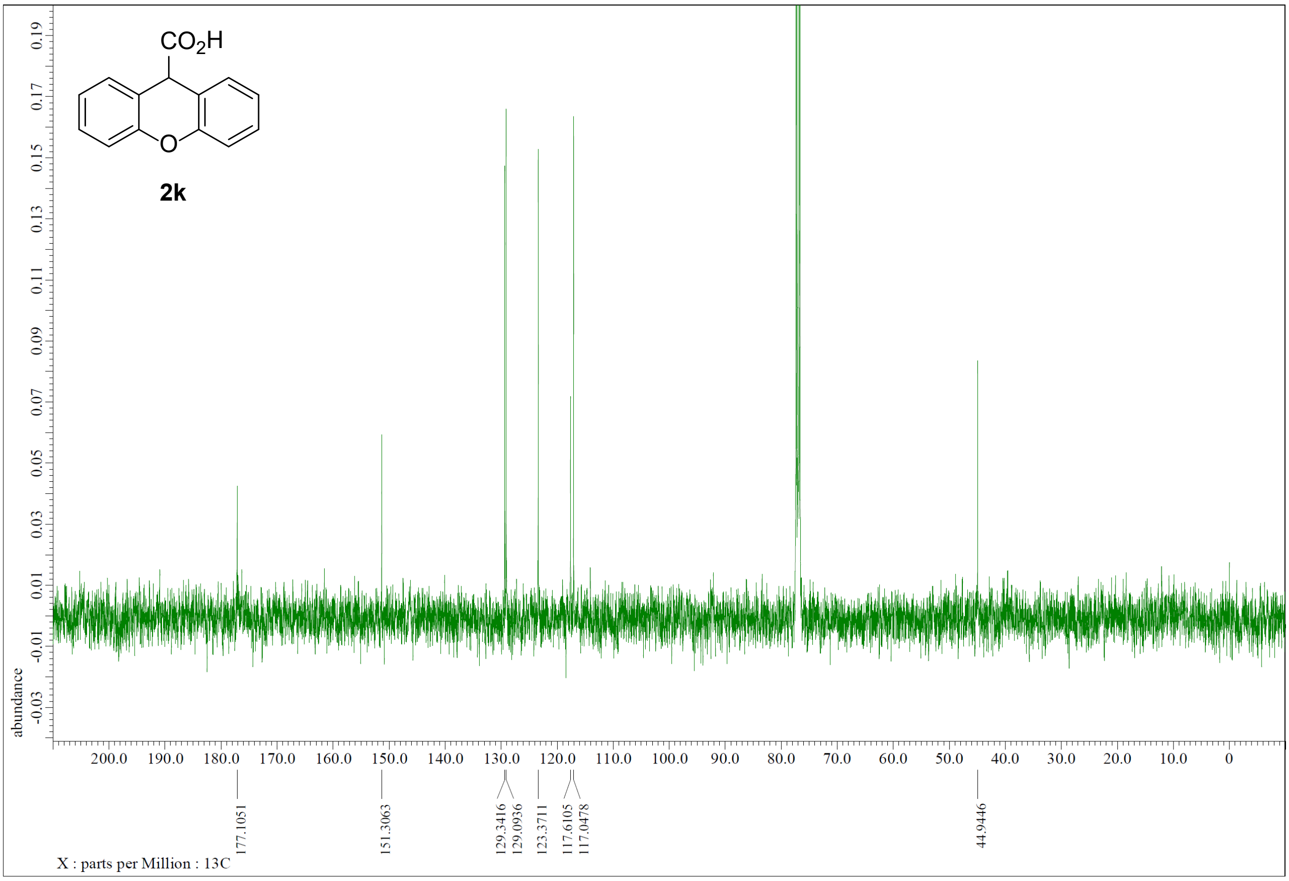

Fig. 18. ^{13}C NMR (100 MHz; $CDCl_3$) spectrum of 9H-xanthene-9-carboxylic acid (**2k**).

10. ^{1}H and ^{13}C spectra of compound 2l.

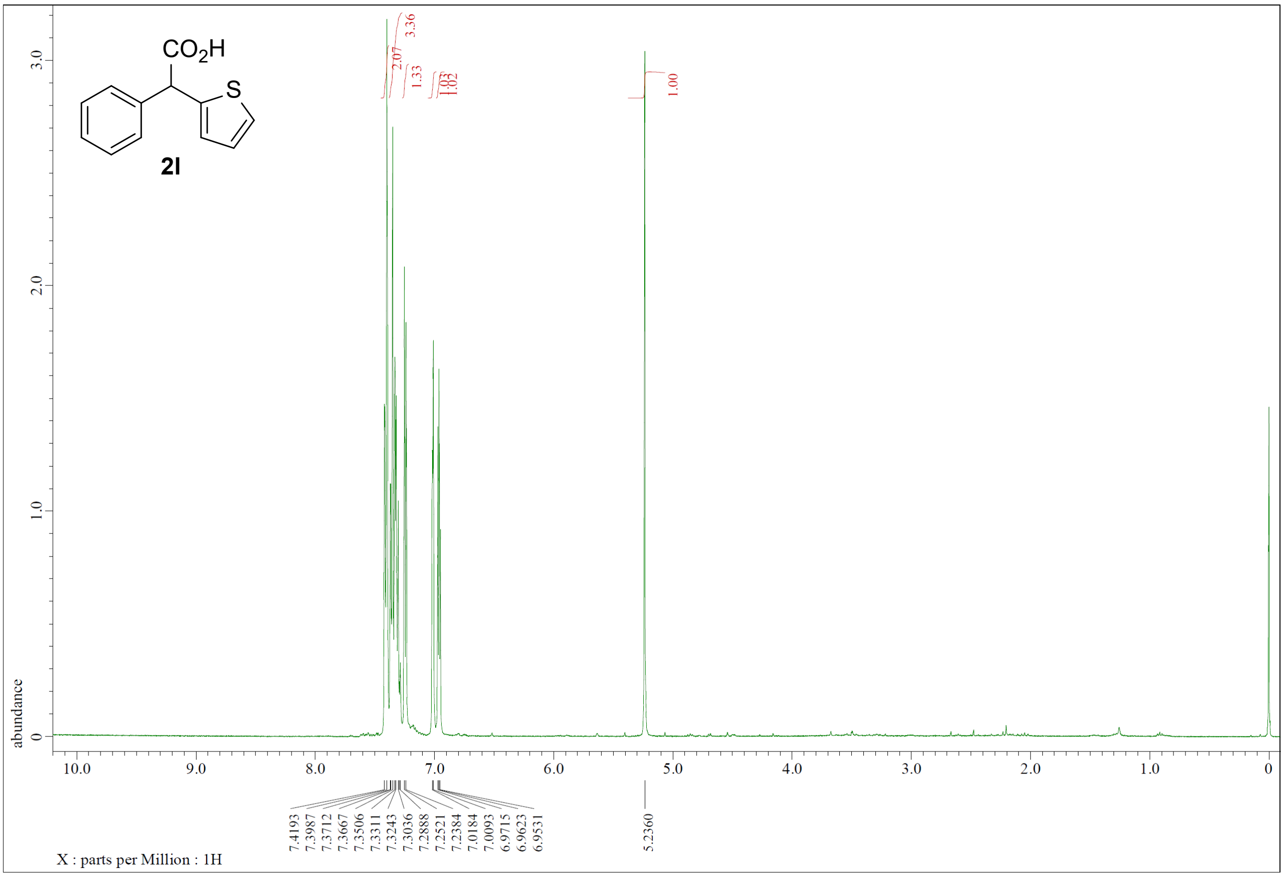

Fig. 19. ^{1}H NMR (400 MHz; $CDCl_3$) spectrum of phenyl(thiophen-2-yl)acetic acid (**2l**).

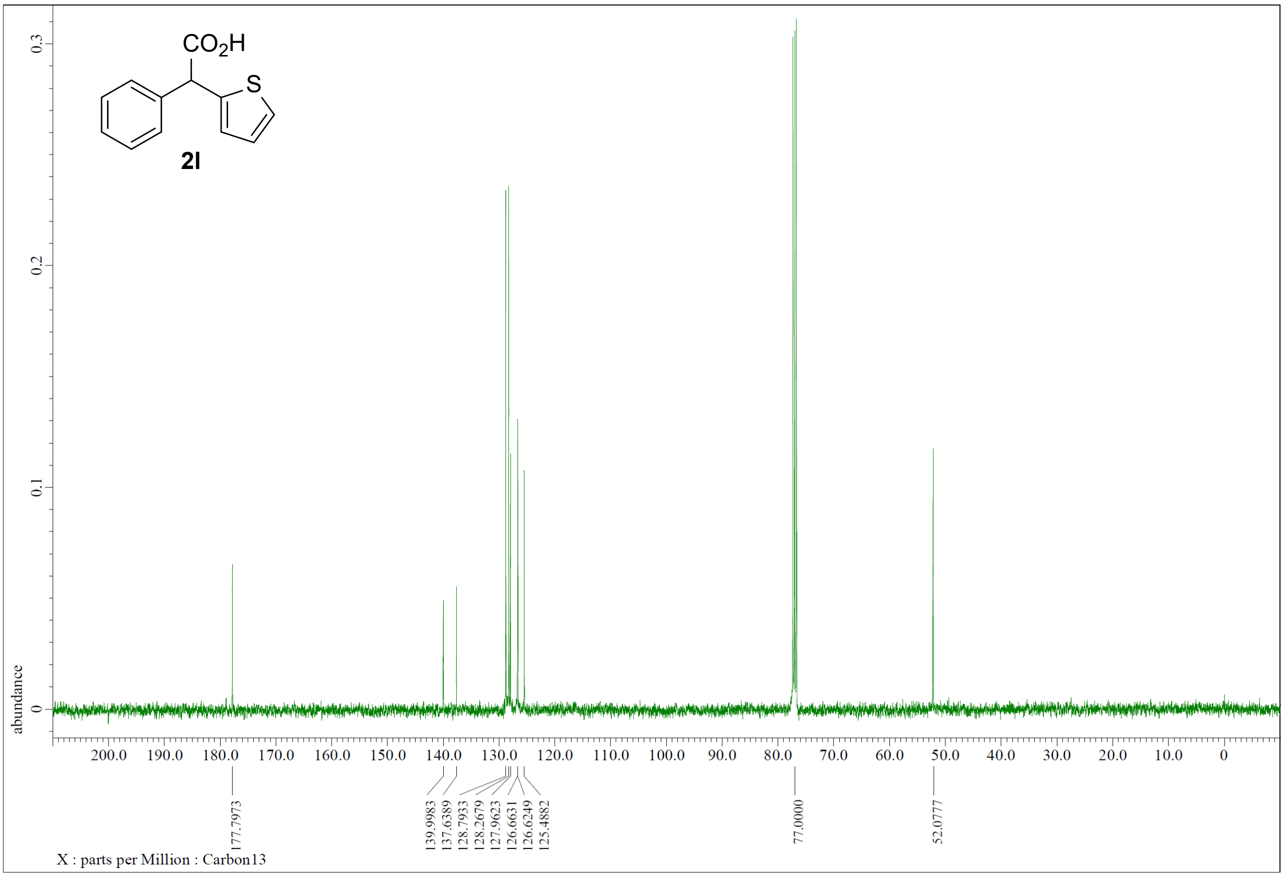

Fig. 20. ^{13}C NMR (100 MHz; $CDCl_3$) spectrum of phenyl(thiophen-2-yl)acetic acid (**2l**).
